# Risk Factors for Intracranial Haemorrhage in Accidents Associated with the Shower or Bathtub

**DOI:** 10.1371/journal.pone.0141812

**Published:** 2015-10-29

**Authors:** Thomas C. Sauter, Jannes Kreher, Meret E. Ricklin, Dominik G. Haider, Aristomenis K. Exadaktylos

**Affiliations:** Department of Emergency Medicine, Inselspital, University Hospital Bern, Bern, Switzerland; University of New South Wales, AUSTRALIA

## Abstract

**Background:**

There has been little research on bathroom accidents. It is unknown whether the shower or bathtub are connected with special dangers in different age groups or whether there are specific risk factors for adverse outcomes.

**Methods:**

This cross-sectional analysis included all direct admissions to the Emergency Department at the Inselspital Bern, Switzerland from 1 January 2000 to 28 February 2014 after accidents associated with the bathtub or shower. Time, age, location, mechanism and diagnosis were assessed and special risk factors were examined. Patient groups with and without intracranial bleeding were compared with the Mann-Whitney U test.The association of risk factors with intracranial bleeding was investigated using univariate analysis with Fisher's exact test or logistic regression. The effects of different variables on cerebral bleeding were analysed by multivariate logistic regression.

**Results:**

Two hundred and eighty (280) patients with accidents associated with the bathtub or shower were included in our study. Two hundred and thirty-five (235) patients suffered direct trauma by hitting an object (83.9%) and traumatic brain injury (TBI) was detected in 28 patients (10%). Eight (8) of the 27 patients with mild traumatic brain injuries (GCS 13–15), (29.6%) exhibited intracranial haemorrhage. All patients with intracranial haemorrhage were older than 48 years and needed in-hospital treatment. Patients with intracranial haemorrhage were significantly older and had higher haemoglobin levels than the control group with TBI but without intracranial bleeding (p<0.05 for both).In univariate analysis, we found that intracranial haemorrhage in patients with TBI was associated with direct trauma in general and with age (both p<0.05), but not with the mechanism of the fall, its location (shower or bathtub) or the gender of the patient. Multivariate logistic regression analysis identified only age as a risk factor for cerebral bleeding (p<0.05; OR 1.09 (CI 1.01;1.171))

**Conclusion:**

In patients with ED admissions associated with the bathtub or shower direct trauma and age are risk factors for intracranial haemorrhage. Additional effort in prevention should be considered, especially in the elderly.

## Introduction

There has been little research on bathroom accidents. For most people, taking a shower or having a bath is just a matter of personal preference. It is unknown whether Emergency Department (ED) admissions associated with the shower or bathtub differ with respect to injury patterns and whether there are special risk factors for traumatic brain injury.

To our knowledge, only one study—in 2008 from the USA—has described the epidemiology of non-fatal bathroom injuries in adults. This showed that most injuries were caused by falls and that a third of the injuries were associated with the shower or bathtub [1]. The incidence of injuries increased with age. The same study group investigated unintentional non-fatal bathroom injuries in children. Also for children most injuries where associated with showering or bathing and were attributed to falls [2].Another study in children employed representative national data from the USA for children and found a peak of incidence in shower/bathtub-related injuries in children younger than 5 years [3].

Although the rate of significant intracranial bleeding in patients with minor head injury is less than 1%, a significant number of patients with head injury undergo radiological imaging of the head [4–7]. The Canadian CT Head rule is a well evaluated decision rule in mild traumatic brain[4,8]. This clinical decision rule defines special “dangerous mechanisms”, but does not consider the impact of special locations, such as bathrooms, which may be especially dangerous when they are wet and slippery.

In the present study, we describe the characteristics of patients admitted to the emergency department for injuries associated with the bathtub or shower. We tested whether there were special injury patterns or special risk factors for traumatic brain injury or intracranial bleeding.

## Data, patients and methods

This retrospective cross-sectional study included all 450,000 patients admitted to our emergency department, the only Level 1 trauma centre in the Canton Bern, Switzerland, from 1 January 2000 to 28 February 2014. Medical data was screened for “bathtub” or “shower”. Data were collected on age, gender, nationality, time of admission, mechanism of injury, final diagnosis, hospital admission, necessity for blood testing and X rays, medication on admission, initial Glasgow Coma Scale (GCS) and initial treatment. Age, GCS, creatinine, sodium, potassium, glucose and haemoglobin were compared for the patients with and without cranial haemorrhage using the Mann-Whitney U test. We calculated power post-hoc for the three most relevant variables observed in this study, namely age, hemoglobin and GCS. Associations between intracranial bleeding and direct trauma, mechanism of fall, gender and accident locations (bathtub or shower) were tested by univariate analysis with Fisher's exact test. Logistic regression was used to test for associations between intracranial bleeding in patients with TBI and the parameters age, sodium, potassium, creatinine, glucose and haemoglobin. Multivariate logistic regression analysis was performed to analyse the effects on cerebral bleeding of any significantly associated variables found in the preceding tests.

For the present study of emergency admissions the protocol was accepted by the Ethics Committee of the Canton Bern, Switzerland. The Ethics Committee of the Canton Bern, Switzerland furthermore waived the need to obtain informed consent for the present study due to the retrospective design. The analysis was done with anonymized data.

All calculations were performed with the SPSS Statistics 21 (IBM Schweiz, Zuerich, Switzerland). The authors performing the data analysis were neither involved in treatment nor data acquisition. A two tailed p value of <0.05 was considered to be statistically significant.

## Results

**Fig 1:**
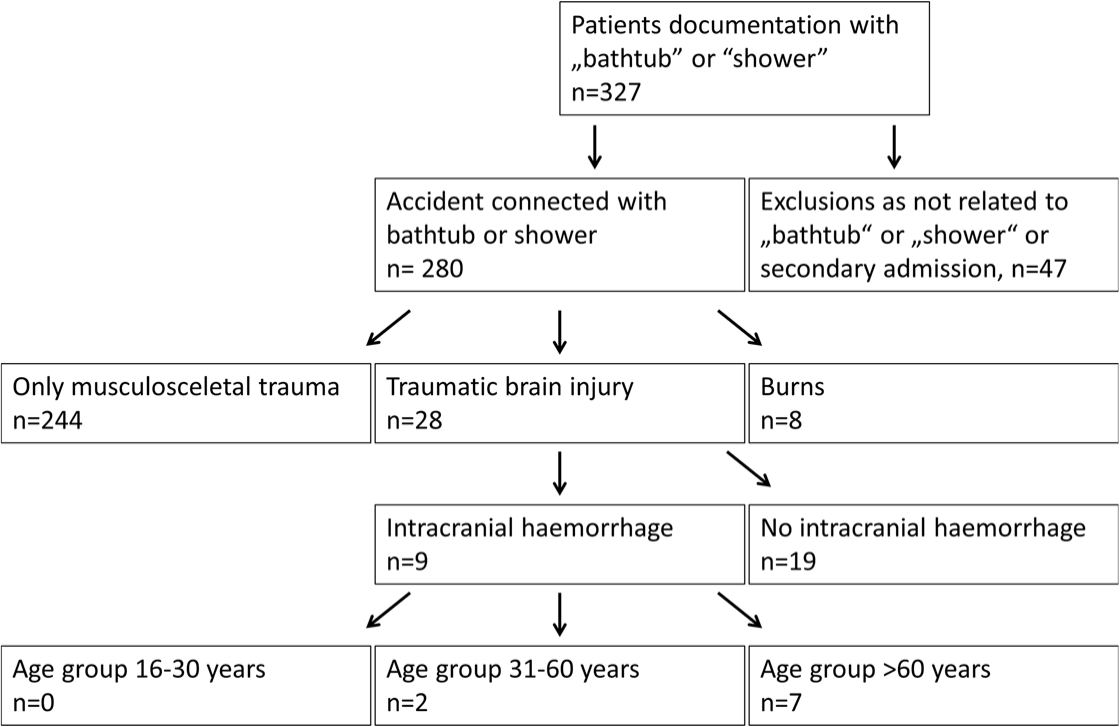
Flowchart. Patient flowchart.

“Bathtub” or “shower” were found in the records of 327 patients “Fig 1”. Forty (40) patients were excluded, as their admission was not related to the bathtub or shower. Seven (7) patients were excluded to minimise selection bias because of secondary transfers from smaller hospitals. The characteristics of the 280 included patients are given in Table 1.

**Table 1:** Characteristics of all patients with bathroom/shower associated accidents; n = 280.

Parameter	Number of Patients
**Mean age in years** (Interquartile range)	48 (31;60)
**Age groups**	
16–30 years	68 (24.3%)
31–60 years	140 (50.0%)
>60 years	72 (25.7%)
**Gender** (male; female)	142 (50.7%); 138 (49.3%)
**Location of Incident**	
Bathtub (%)	168 (60.0%)
Shower (%)	112 (40.0%)
**Accident location at home**	266 (95.0%)
**Time of Incident**	
6am–11am	57 (20.4%)
11am–4pm	15 (5.4%)
4pm–10pm	49 (17.5%)
10pm–6am	20 (7.1%)
Unknown	134 (47.9%)
**Investigations**	
X-ray	238 (85.0%)
Blood testing	80 (28.6%)
**Emergency surgery**	29 (10.4%)
**Hospitalisation**	65 (23.2%)
**Indications for cranial computer tomography in patients with traumatic brain injury (multiple answers possible)**	28 (10%)
GCS<15 at 2 h after injury	5 (1.8%)
Suspected open or depressed skull fracture	1 (0.4%)
Sign of basal skull fracture	2 (0.7%)
Vomiting (≥two episodes)	4 (1.4%)
Age ≥65 years	11 (3.9%)
Amnesia before impact >30 min	1 (0.4%)
Dangerous mechanism	0 (0%)
Post-traumatic seizure	3 (1.1%)
Focal neurological deficit	6 (2.1%)
Intoxication	2 (0.7%)
Medication (Marcumar)	5 (1.8%)
Indication unknown	3 (1.1%)

One hundred and sixty-eight (168) accidents (60.0%) took place in the bathtub and 112 (40.0%) in the shower. Ninety-eight (98) (84.5%) of shower and all bathtub accidents took place in a domestic area. In 27 patients (9.6%), the accident took place because of a medical problem; 235 patients suffered a direct trauma by hitting an object (83.9%) and in 185 patients (66.1%) the cause of the accident was a fall. In 149 patients (53.2%) the reason for falling was slipping. Overall 29 patients (10.4%) had to undergo emergency surgery. A fracture was found in 63 patients (22.5%) and 52 (18.6%) patients suffered lacerations. Further details about time of incident and necessary investigations are provided in Table 1.

### Traumatic brain injury

Traumatic brain injury occurred in 28 (10.0%) patients. Table 1 shows the indications for the cranial computer tomography (CCT) scan in these patients. Apart from one patient with severe traumatic brain injury (GCS 3 at admission, 3.6% of TBI), all patients had minor traumatic brain injury (defined as GCS 13–15) at admission.

All patients with intracranial bleeding were older than 48 years (mean 72±11 years, Flowchart 1).

One patient needed emergency neurosurgical intervention. All of the patients with intracranial haemorrhage had to stay in hospital for surveillance (n = 9).

In comparison to patients with TBI but without intracranial bleeding, patients with intracranial haemorrhage were significantly older and had higher haemoglobin levels (p<0.05 for both, Table 2). No other significant difference between the groups was detected (Table 2). The post-hoc calculated power for the three most relevant variables observed in this study, namely age, hemoglobin and GCS were 95.6% (age), 68.5% (haemoglobin) and 46.23% (GCS).

**Table 2:** Comparison of patients with traumatic brain injury with (n = 9) bleedingand patients without intracranial bleeding (n = 271).

Parameter	Intracranial bleeding	No intracranial bleeding	p
**Age**	72±11*	47±19*	**0.002**
GCS	13.4±3.9	14.9±0.35	0.332
Sodium	138±5	132±12	0.336
Potassium	4.0±0.4	3.7±0.5	0.335
Creatinine	60±30	80±13	0.127
Glucose	7.2±1.7	19.2±19.4	0.110
**Haemoglobin**	135.6±14.8*	121.5±15.9*	**0.046**

Mean; ±Standard deviation, Mann-Whitney U, p<0.05*.

Univariate analysis showed that direct trauma and age were associated with intracranial haemorrhage in patients with TBI (both p<0.05, Table 3). There was no significant association with the mechanism of fall, the location of the fall (bathtub or shower) or gender (Table 3).

**Table 3:** Associations of intracranial bleeding in patients with TBI with different parameters in univariate analysis. Fisher's exact test, p<0.05*

Parameter	p
Gender	0.103
Location shower	0.657
Location bathtub	0.687
**Direct trauma**	0.020*
Fall	0.114

In the univariate logistic regression analysis, age was significantly associated with cerebral bleeding (p = 0.002, Table 4). In patients with TBI, intracranial bleeding was not associated with the parameters sodium, potassium, creatinine, glucose or haemoglobin.

Multivariate logistic regression found a significant association between age and cerebral bleeding (p = 0.025), but with no other factor.

**Table 4:** Associations of intracranial bleeding in patients with TBI with different parameters in univariate analysis. Logistic regression, p<0.05*

Parameter	p	Odds ratio (confidence interval)
**Age**	0.012*	**1.091 (1.02;1.171)**
Sodium	0.246	1.098 (0.937;1.287)
Potassium	0.296	4.759 (0.254;8.984)
Creatinine	0.199	1.050 (0.975;1.132)
Glucose	0.453	0.771 (0.391;1.521)
Haemoglobin	0.137	1.069 (0.979;1.164)

## Discussion

Our study showed that the vast majority of bathroom-related accidents are associated with direct trauma by hitting an object, mostly due to slipping and falling. Many patients with TBI suffer intracranial bleeding. Patients sustaining intracranial bleeding are older and have a significantly higher haemoglobin concentration in group comparison. In our population, the risk factors for intracranial haemorrhage in patients with TBI were direct trauma and age.

Nearly 30% of our patients with mild TBI sustained intracranial bleeding. In contrast, Stiell et al. (2001) found that 8% of their patients suffered clinically important intracranial haemorrhage and 1% needed neurosurgical intervention [8]. It might be thought that our higher values indicate selection bias in our Level 1 trauma and stroke centre. On the other hand, the authors also found only 4.8% intracranial bleeding in patients with mild TBI in a non-selected population from our department in 2013 [9]. The difference may be explained by the fact that older people fall more often and more severely in the bathtub or shower, as bathrooms are known to be the leading location of falls at home in elderly people [10]. The main risk factor for intracranial bleeding that we could identify in our study was age. This is consistent with the literature describing age>65 as predictor for clinically important brain injury [4,8,10]. In our population, no intracranial bleeding was reported in patients <50 years of age.

In patients with mild TBI (GCS 13–15), the clinician's decision to obtain a cranial CT-scan may be difficult and should be based on decision rules with good evidence, such as the Canadian CT-head rule or the New Orleans Criteria. This is particularly true for the high risk population of elderly patients in the bathroom [10]. Therefore it is important to remember that all intracranial haemorrhages in our study population were assessed and diagnosed by the Canadian CT-Head rule or were linked to new focal neurological deficits or oral anticoagulants, which are specifically excluded from the Head Rule. The reason why there is no significant GCS difference between the groups with and without bleeding, although this might be expected, may be the missing power.

We found significantly higher haemoglobin values in our group with intracranial bleeding than in the control group with TBI and no intracranial haemorrhage. It is already known that haemoglobin levels may increase after taking a bath, perhaps due to dehydration—particularly when the bathroom is hot [11]. As this study was retrospective, this cannot be further investigated.

Our study showed that the vast majority of patient admissions associated with the bathtub or shower were due to direct trauma. Most of the patients slipped and suffered a fall. Falls are known to be the leading cause of unintentional injury in adults of 65 years or older [9]. Nearly 40,000 people are treated annually in EDs because of falls in the USA. 66.8% percent of our patients had suffered a fall. This is consistent with Rosen et al. (2013), who found that the bathroom was the most common location of falls at home (35.7%) [10].

As advanced age is a risk factor for intracranial bleeding, we consider that elderly people should be informed about the potentially serious threat in their bathrooms, as well as being provided with more technical aids and human support. It must be born in mind that elderly people are highly susceptible to major injuries after a fall [12,13].

### Limitations

The main limitation to our study is the risk of selection bias, as our hospital is not only a Level 1 trauma centre, but also a stroke centre. To minimise this bias, patients arriving by secondary transport from external hospitals were excluded. The data was collected retrospectively and therefore could not be balanced. Furthermore, the presented data are recovered from a large database and not collected by the authors; there is no guarantee that all bathroom related injuries were documented and found.

In summary we found, that elderly patients, patients suffering direct trauma by hitting an object are specifically at risk of intracranial bleeding in accidents associated with the bathtub and shower. As the incidence of intracranial bleeding in elderly people is high in our population, further efforts should be made to prevent these accidents.
